# Production of ^13^C-labeled docosahexaenoic acid from heterotrophic marine microorganisms *Aurantiochytrium mangrovei* and *Crypthecodinium cohnii* enabling fluxomic applications

**DOI:** 10.3389/fbioe.2025.1690863

**Published:** 2025-11-19

**Authors:** Manon Buscaglia, Bastian Gouriou, Yoann Asquoët, Nelly Le Goïc, Fabienne Le Grand, Mayssa Hachem, Philippe Soudant

**Affiliations:** 1 Univ Brest, CNRS, IRD, Ifremer, LEMAR, Plouzané, France; 2 Department of Chemistry, Khalifa University of Sciences and Technology, Abu Dhabi, United Arab Emirates; 3 Food Security and Technology Center, Khalifa University of Sciences and Technology, Abu Dhabi, United Arab Emirates

**Keywords:** 13C-labeled DHA, *Aurantiochytrium mangrovei*, *Crypthecodinium cohnii*, omega-3, sustainable production

## Abstract

Docosahexaenoic acid (DHA, 22:6n-3) is the predominant polyunsaturated fatty acid in the human brain and eyes, playing a crucial role in vision and cognitive development. DHA deficiency has been associated with ocular diseases, such as macular degeneration and glaucoma, as well as neurodegenerative disorders. Since the human body has a limited ability to synthesize DHA from its precursor, alpha-linolenic acid (ALA, 18:3n-3), targeted DHA supplementation is essential for these patients. To investigate DHA metabolism and integration, researchers commonly use stable (^2^H,^13^C) or radioactive (^3^H,^14^C) isotopes, which are expensive and not widely accessible, restricting the scope and duration of studies. This study aimed to develop a sustainable method for biosynthesizing uniformly labeled ^13^C-DHA by culturing the heterotrophic protists *Aurantiochytrium mangrovei* and *Crypthecodinium cohnii* with ^13^C-glucose. The major fatty acids (FA) of *A. mangrovei* included 16:0, 22:5n-6 (DPA), and DHA, with DHA accounting for 50.5% ± 4.9% of the total FA. Gas Chromatography-Mass Spectrometry (GC-MS) analysis revealed a^13^C-enrichment of DHA at 96.7% ± 0.4% after the effective High Performance Liquid Chromatography (HPLC) purification. The predominant FA of *C. cohnii* were 12:0, 14:0, 16:0, and DHA, with DHA representing around 27% of the total FA and exhibiting a^13^C-enrichment of 86.3% ± 1.6%. Based on FA content, *A. mangrovei* showed a balanced distribution of neutral and polar lipids, with DHA predominantly in the polar fraction (57.8% ± 3.1%), whereas *C. cohnii* exhibited a predominance of neutral lipids (82.4% ± 0.3%), which contained the majority of its DHA (57.5% ± 1.0%).

## 1 Introduction

Docosahexaenoic acid (DHA, 22:6n-3), an essential omega-3 long-chain polyunsaturated fatty acid (LC-PUFA), is primarily found in human’s brain and eyes, where it plays key roles in neuroprotection and offers various nutraceutical benefits (Hachem et al., 2020a). Within the eye, DHA is crucial for promoting the proliferation of corneal epithelial cells and the regeneration of nerves after inflammation (He and Bazan, 2010), while also helping to prevent corneal neovascularization (Sapieha et al., 2011; Connor et al., 2007). This inhibition reduces both corneal opacity and microvascular damage, making DHA beneficial for managing post-surgical complications and more complex conditions like diabetes and metabolic syndrome. As populations aging, Alzheimer’s disease (AD) is becoming an increasing concern worldwide, with research showing reduced DHA levels in the brain of affected patients. Because the body has a limited ability to biosynthesize DHA from its precursor alpha-linolenic acid (ALA, 18:3n-3), dietary intake of DHA is crucial to compensate for this deficiency and support brain health. In the context of prevention and potential treatment of neurodegenerative disease, particularly AD, researchers are investigating the way different forms of DHA, such as non-esterified DHA and DHA esterified in phospholipids and triglycerides (TG), influence the brain’s bioavailability (Hachem et al., 2020b; Hachem et al., 2016; Thiès et al., 1992; Thiès et al., 1994; Bernoud et al., 1999). For this purpose, labeled molecules (stable or radioactive isotopes) were used in *in vitro*, *in vivo*, and *ex vivo* studies to follow the DHA’s metabolism and conversion in the body.

Several techniques have already been developed in order to examine the natural variations in ^13^C/^12^C ratio and trace n-3 PUFA metabolism (Metherel et al., 2019). However, tracing these metabolites can be challenging when the ^13^C/^12^C signatures of different compounds are too similar. In such cases, uniformly labeled ^13^C-DHA offers a powerful tool for precise analysis of DHA distribution, metabolism, and conversion into other metabolites. Unlike radioactive tracers, stable isotope applications like ^13^C-DHA pose no safety risks, making them ideal for human metabolism studies.

Although stable isotopes have different atomic masses, they are chemically identical. Therefore, ^13^C-labeled lipids behave biochemically similarly to their non-labeled equivalents, allowing for accurate tracking of lipid metabolism (Triebl and Wenk, 2018). Indeed, isotopically labeled substrates are incorporated into organic macromolecules such as lipids, carbohydrates, and proteins, enabling researchers to identify metabolic intermediates or final products such as fatty acids (FA). This allows for the quantification of labeling levels and provide insights into biosynthesis pathways, offering a deep understanding of lipid regulation and metabolism in various research models (Schlame et al., 2020).

However, in clinical nutrition studies, where ^13^C-DHA is administered orally or intravenously, large amounts of this expensive tracer (at least 40 mg per individual) may be required for effective labeling. Furthermore, injectable tracers used in clinical trials must be sterile, adding more to the complexity and cost. In human, ^13^C-labeled metabolic flux analysis (^13^C-MFA) has been applied in limited clinical studies. For instance, one study involving elderly participants (aged 60–70) investigated the bioavailability and metabolic flux of ^13^C-DHA following oral intake of a 50 mg dose of ^13^C-DHA, either in non-esterified form, esterified in a structured phospholipid (^13^C-AceDoPC®), or esterified in triglycerides (^13^C-TG) (Hachem et al., 2020b).

The availability of sufficient amounts of uniformly labeled ^13^C-DHA is critical for studying its metabolic fate. Unfortunately, ^13^C-DHA is prohibitively expensive and often unavailable, with costs exceeding $2,000 for just 5 mg. The scarcity of uniformly labeled ^13^C-DHA explains its high market price, as seen with suppliers like Cambridge Isotope Laboratories (CIL). Additionally, commercially available ^13^C-DHA is only sold as an ethyl ester or a methyl ester, limiting its utility. To advance research, it is essential to develop cost-effective methods for producing ^13^C-DHA in various natural lipid forms, such as phospholipids including phosphatidylcholine (PC), phosphatidylethanolamine (PE), phosphatidylserine (PS), phosphatidylglycerol (PG), lysophosphatidylcholine (Lyso-PC) as well as triglycerides (TG), for use in both clinical nutrition studies and biological research. This situation stresses the need for alternative methods to study DHA metabolism, particularly its accumulation in the brain.

One promising approach is to produce ^13^C-labeled LC-PUFA using microorganisms in photobioreactors or fermenters supplied with a^13^C-labeled source, whether ^13^C-CO_2_ or ^13^C-glucose. In this context, studies have shown that ^13^C-MFA can provide valuable insights into lipids’ synthesis and accumulation in phototrophic and heterotrophic microorganisms (Xiong et al., 2010; Martzolff et al., 2012; Cui et al., 2018). Recent researches on marine phytoplankton, such as diatoms, prymnesiophytes, and dinoflagellates, have demonstrated the potential of this method, achieving up to 60% ^13^C-enrichment in LC-PUFA within just 24 h of culture (Remize et al., 2022; Remize et al., 2020a; Remize et al., 2020b). For example, Acién Fernández et al. (2003) developed a system to produce ^13^C-LC-PUFA from *Phaeodactylum tricornutum* using labeled ^13^C-CO_2_ (Acién Fernández et al., 2003). However, the degree of labeling is inverse proportional with the number of carbons; the higher the number of carbons was, the lower was the ^13^C enrichment. For eicosapentaenoic acid (EPA, 20:5n-3), isotopic composition ranged from 36% to 53%, based on the detection method, whether Gas Chromatography-Mass Spectrometry (GC-MS) or Gas Chromatography-combustion-Isotope Ratio Mass Spectrometry (GC-c-IRMS). More importantly, the challenge in producing uniformly labeled ^13^C-LC-PUFA lies in preventing the incorporation of non-labeled ^12^C-CO_2_ during the cultivation process.

In addition to microalgae, heterotrophic microorganisms can also be utilized to biosynthetically produce uniformly labeled ^13^C-lipids using ^13^C-glucose as a carbon source. For example, Rampler et al. (2017) successfully used ^13^C-glucose to produce isotopically labeled lipids in yeast, achieving excellent ^13^C enrichment (>99.5%) for over 200 lipid species (Rampler et al., 2017). While yeast is easy to label, it does not produce LC-PUFA such as DHA or EPA, which limits its application in DHA research.

One significant challenge is the cultivation of DHA-rich heterotrophic protists while monitoring ^13^C enrichment in lipids throughout the process. For example, *Schizochytrium sp.*, a DHA-producing thraustochytrid, was cultured with ^13^C-sodium acetate, achieving over 97% ^13^C incorporation in DHA (Watanabe et al., 2000). Similarly, *Hyalochlorella marina*, grown with glutamate as a nitrogen source, produced ^13^C-labeled LC-PUFA (EPA, DPA n-3, n-6, and DHA) with up to 90% ^13^C incorporation in DHA (Le et al., 2007). DHA could not achieve a higher level of labeling due to the potential incorporation of non-labeled carbon from the glutamate into its structure. In a more recent study, *Crypthecodinium cohnii (C. cohnii)* was grown on a defined medium supplemented with uniformly labeled ^13^C-glucose, achieving a maximum ^13^C enrichment of 96.8% for DHA (Song et al., 2020). However, non-labeled carbon from glutamate was likely incorporated, reducing the overall ^13^C enrichment. These findings emphasized on the need for cultivating heterotrophic protists with only uniformly labeled ^13^C-glucose as the carbon source.

The actual study has been conducted using *Aurantiochytrium mangrovei (A. mangrovei)* cultured on a medium designed specifically for ^13^C lipid enrichment. This medium contained only uniformly labeled ^13^C glucose as the carbon source, while other nutrients like nitrogen were provided in inorganic forms to prevent dilution from organic compounds. A characteristic of *A. mangrovei* is its ability to produce docosapentaenoic acid (DPA, 22:5n-6). This LC-PUFA has the drawback of structural similarities to DHA, which can lead to its assimilation in place of DHA, without providing the same functional benefits (Lim et al., 2005). The differentiation between DHA and DPA n-6 is often challenging, and this study aimed to achieve their precise separation through High Performance Liquid Chromatography (HPLC). Another approach has been applied to *C. cohnii*, cultured on a simpler medium containing yeast extract and ^13^C-glucose, which contains a higher proportion of DHA. Furthermore, it may offer different DHA-rich lipid species compared to *A. mangrovei*, and *C. cohnii* does not produce DPA n-6, which may simplify DHA purification. A particular attention was given, for the first time in this type of research and these organisms, to the ^13^C-enrichment of FA within neutral and polar lipid fractions. This innovation forms the basis of the proposed research, which aims to develop sustainable method for producing uniformly labeled ^13^C-DHA through the cultivation of heterotrophic protist *A. mangrovei* and dinoflagellate *C. cohnii*.

## 2 Materials and methods

### 2.1 Materials

U-^13^C-glucose was purchased from Cambridge Isotopes Laboratories (Andover, MA, United States). Sodium chloride (NaCl) was obtained from FisherScientific, Cobalt (II) chloride hexahydrate (CoCl_2_·6H_2_O) from BHD Lab, Copper (II) sulfate pentahydrate (CuSO_4_·5H_2_O) from Fluka, Monopotassium phosphate (KH_2_PO_4_), sulfuric acid (H_2_SO_4_) and Yeast extract from VWR, Manganese (II) chloride tetrahydrate (MnCl_2_·4H_2_O) and sodium hydroxide (NaOH) from Carlo Erba Reagents, Folate from Alfa Aesar, and Ascorbic acid and DNS (3,5-dinitrosalicylic acid) from Acors organic. Ammonium sulfate ((NH_4_)_2_SO_4_), Calcium chloride dihydrate (CaCl_2_·2H_2_O), Iron (II) sulfate heptahydrate (FeSO_4_·7H_2_O), ^12^C-glucose, Magnesium sulfate heptahydrate (MgSO_4_·7H_2_O), and Thiamin were received from Applichem. Sodium molybdate dehydrate (Na_2_MoO_4_·2H_2_O), and Zinc sulfate heptahydrate (ZnSO_4_·7H_2_O) were purchased from Merck. Sodium selenite (Na_2_SeO_3_), Vitamins B and B12, Niacin, Potassium sodium tartrate tetrahydrate (C_4_H_4_KNaO_6_·4H_2_O), potassium hydroxide (KOH) and organic solvents (acetone, chloroform, Hexane, and Methanol) were received from Sigma-Aldrich.

### 2.2 Media and culture conditions

#### 2.2.1 Aurantiochytrium mangrovei

*Aurantiochytrium mangrovei* (RCC893) was grown in a synthetic medium developed by our laboratory using glucose as the only source of organic carbon. The medium contained per liter: 25.03 g NaCl, 9.44 g (NH_4_)_2_SO_4_, 0.76 g CaCl_2_·2H_2_O, 0.013 mg CoCl_2_·6H_2_O, 1.97 mg CuSO_4_·5H_2_O, 20 mg FeSO_4_·7H_2_O, 10 g glucose, 1.98 g KH_2_PO_4_, 0.49 g MgSO_4_·7H_2_O, 4.69 mg MnCl_2_·4H_2_O, 0.12 mg Na_2_MoO_4_·2H_2_O, 0.037 mg Na_2_SeO_3_, 17.59 mg ZnSO_4_·7H_2_O, 0.14 mg thiamin, 0.19 mg vitamin B, 1.051 mg niacin, 0.20 mg folate, 7.37 mg ascorbic acid, and 0.016 mg vitamin B12. The medium was sterilized by filtration through 0.22 μm aPES filters (Nalgene, ThermoScientific) and the pH was adjusted to 6.5 with NaOH (3 M). Culture was carried out in triplicate at 20 °C under stirring at 100 rpm. In this study, two conditions were tested: one using ^13^C-labeled glucose to generate a “^13^C-enriched” condition, and the other using unlabeled ^12^C-glucose as a “Control” condition, in order to assess the efficiency of isotopic enrichment under comparable culture conditions. Culture was conducted in two phases over 15 days. Firstly, *A. mangrovei* was inoculated at 5 × 10^6^ cells. mL^-1^ in 40 mL of medium in 100 mL sterile flasks, to ensure successful culture and limits the loss of ^13^C-glucose. Subsequently, part of this culture was inoculated at the same cell concentration in 200 mL of medium in 500 mL sterile flasks, following the same culture protocol, to produce more DHA and validate the scale-up potential of the experiment.

Almost daily, 600 µL of medium were collected for controls measuring: (i) cell concentration, (ii) pH, (iii) osmolarity, and (iv) glucose concentration. Once glucose could become limiting, the culture was terminated (arbitrarily set at 1 g.L^-1^). The first-step culture (2 × 15 mL) was filtered at day 5 on pre-combusted (6 h, 450 °C) 47 mm GF/F filters (0.7 µm, VWR®, WhatmanTM), to obtain “A40-ctrl-FL” and “A40-13C-FL”. The second-step culture was collected at day 10, 30 mL were filtered (2 × 20–30 mL) and the remaining volume was centrifuged (5,000 g, 15 min, 15 °C, Eppendorf Centrifuge 5810 R) and freeze-dried (SRK, Lyovac), to obtain respectively “A200-ctrl-FL” and “A200-13C-FL”, and “A200-ctrl-FD” and “A200-13C-FD”. The sample code is explained in Table 1.

**TABLE 1:** Sample codes based on the nomenclature: species - culture volume - culture condition - harvesting method.

Code	Species	Culture volume	Culture condition	Harvesting method
A40-ctrl-FL	*A. mangrovei*	40	Control	Filtration
A40-13C-FL			^13^C-enriched	
A200-ctrl-FL		200	Control	
A200-13C-FL			^13^C-enriched	
A200-ctrl-FD			Control	Freeze-drying
A200-13C-FD			^13^C-enriched	
C40-ctrl-FL	*C. cohnii*	40	Control	Filtration
C40-13C-FL			^13^C-enriched	
C40-ctrl-FD			Control	Freeze-drying
C40-13C-FD			^13^C-enriched	

#### 2.2.2 Crypthecodinium cohnii

*Crypthecodinium cohnii* (CCMP316) was grown in a culture medium, adapted from de Swaaf et al. (2003), containing 5.5 g Yeast extract and 9 g glucose per liter of sterile seawater, and then the medium was filtered following the same procedure as the one mentioned for *A. mangrovei* (de Swaaf et al., 2003). This medium differed from the one used for *A. mangrovei* in order to ensure compatibility with *C. cohnii*. It was also simpler to produce, which could be an advantage if *C. cohnii* is able to synthesize ^13^C-labeled DHA under these conditions, particularly in terms of large-scale production.*C. cohnii* was inoculated at 5 × 10^4^ cells. mL^-1^ in 40 mL of medium in 100 mL sterile flasks under the same conditions as *A. mangrovei*. The same parameters were examined and the culture was collected at day 12. Then, 10 mL were filtered and 25 mL were centrifuged and freeze-dried, to obtain “C40-ctrl-FL” and “C40-13C-FL”, and “C40-ctrl-FD” and “C40-13C-FD”. The sample code is explained in Table 1.

### 2.3 Culture monitoring

The concentration and cellular parameters (size, complexity, and lipid content) were assessed using a Guava flow cytometer (Easy-Cyte 5HT, Luminex) equipped with a 488 nm blue laser, forward scatter (FSC) and side scatter (SSC) detectors, and three fluorescence detectors: green (525/30 nm), yellow (583/26 nm) and red (695/50 nm). Cell populations were identified through cell relative size obtained by FSC value and cell complexity obtained by SSC value according to Lelong et al. (2011) (Lelong et al., 2011). The flow cytometry measurements were performed at a flow rate of 59 μL min^-1^ on samples diluted by 100 or 1,000.

The lipid content was analyzed using the BODIPY fluorescent probe (BODIPY 493/503, Invitrogen, Eugene OR, United States, final concentration of 10 µM), which stains lipid reserves and emits green fluorescence proportional to lipid content in cells according to Lelong et al. (2011) (Lelong et al., 2011). The cell’s concentration was expressed in cells per mL, and cellular parameters including FSC, SCC, and lipid reserve were expressed in arbitrary units (A.U.). All cytometric analyses were performed in duplicate.

On daily basis, pH was monitored with a pH meter (Mettler Toledo, SevenCompactTM S210) in order to adjust it to 6-7 with NaOH (3 M) and to reduce the impact on the cells.

Additionally, the osmolarity was measured from 50 µL of culture with an osmometer (Gonotec, Osmomat 3,000) after a few days to check its stability at approximately 1,000 mOsmol.kg^-1^.

For glucose, the concentration was determined using spectrometric titration with 3,5-dinitrosalicylic acid (DNS). Indeed, DNS is reduced by glucose to 3-amino-5-nitrosalicylic acid, a red compound whose color intensity is proportional to glucose concentration (Miller, 1959; Beidler et al., 2024). Regularly, 500 µL of culture were centrifuged (5,000 g, 15 min, 15 °C) to remove cells. The supernatant was diluted by a factor of 10 with milli-Q water. In a tube, 1 mL diluted sample and 1 mL DNS mixture (containing per 1 L milli-Q water: 10 g DNS, 300 g C_4_H_4_KNaO_6_·4H_2_O and 16 g NaOH) were mixed and placed in a water bath at 95 °C for 10 min. After cooling to room temperature, 7.5 mL of milli-Q water were added before reading the absorbance at 540 nm (UV-1700 Pharmaspec, UV-VIS spectrophotometer, Shimadzu). Finally, the glucose concentration was obtained in comparison with a standard glucose range (1–5 g.L^-1^).

### 2.4 Lipid extraction

All manipulations were carried out using glassware previously calcined (450 °C, 6 h) or cleaned with acetone. The filter or 20 mg of homogenized freeze-dried sample were placed in a vial containing 6 mL of chloroform/methanol mixture (CHCl_3_/CH_3_OH, 2:1, v/v) applying a modified method of Folch et al. (1957) (Folch et al., 1957). Samples were then sonicated 10 min, to enhance lipid extraction, and stirred at 400 rpm for 20 min on a shaking table. Until further analysis, all lipid extracts were stored at − 20 °C under nitrogen atmosphere to avoid oxidation.

### 2.5 Separation of neutral lipids (NL) and polar lipids (PL)

NL and PL fractionation was performed, only on freeze-dried samples, using 1 mL of total lipid extract (TL) evaporated to dryness under nitrogen. The sample was recovered with three washes of 0.5 mL CHCl_3_/CH_3_OH (98:2, v:v) and deposited on silica gel column (40 × 4 mm, silica gel 60Å 63–200 µm rehydrated with 6% H_2_O, 70–230 mesh, Sigma-Aldrich). The first elution was performed with 10 mL CHCl_3_/CH_3_OH (98:2, v:v) to collect NL, the second with 20 mL methanol to collect PL. Both were collected in glass vials containing 2.3 µg internal standard (C23:0, Sigma-Aldrich) (Soudant et al., 2022). All lipids’ fractions were stored at −20 °C under nitrogen until further analysis.

### 2.6 Fatty acid analysis in NL, PL and total lipids (TL)

Fatty acids methyl esters (FAME) were obtained according to the protocol adapted from Le Grand et al. (2014) (Le Grand et al., 2014). The treatment was carried out on NL, PL, and TL fractions (for the latter, 1 mL was used and supplemented with the internal standard C23:0, as in the previous fractions). Briefly, after evaporation to dryness of the TL fractions, saponification was completed by adding 1 mL KOH-CH_3_OH (0.5 M) to the lipid extract and heated at 80 °C for 30 min. The transesterification was then conducted by adding 1.6 mL H_2_SO_4_/CH_3_OH mixture (3.4%, v:v) and heated at 100 °C for 10 min. Hexane (0.8 mL) and distilled water saturated with hexane (1.5 mL) were added. After homogenization and centrifugation (738 g, 1 min, SorvallTM ST16R, ThermoScientific), the lower CH_3_OH/H_2_O phase was discarded. Hexane fraction containing FAME was washed twice more with distilled water (1.5 mL). All samples were stored at −20 °C under nitrogen until further analysis.

FAME composition analysis was conducted using Gas Chromatography coupled to Flame Ionization Detector (GC-FID, Trace 1,300, ThermoScientific). 2 μL of sample was injected at 250 °C in splitless mode with hydrogen (H_2_) as the gas carrier at 2 mL min^-1^ flow and an oven temperature of 60 °C. The GC was equipped with two FID (280 °C) associated with one column each: a polar (DB-HeavyWax: 30 m × 0.25 mm ID × 0.25 μm, Agilent) and a non-polar column (DB-5: 30 m × 0.25 mm ID × 0.25 μm, Agilent). The oven’s temperature was raised to 150 °C at 50 °C.min^−1^, to 170 °C at 3.5 °C.min^−1^, to 185 °C at 1.5 °C.min^−1^, to 225 °C at 2.4 °C.min^−1^, and finally to 250 °C at 5.5 °C.min^−1^ and maintained for 15 min. FAME’s identification on “Control” samples was obtained by comparison of their retention times with those of external commercial standards (Supelco 37 Component FAME Mix, the PUFA no. 1 and no. 3, Sigma Aldrich) using Chromeleon (7.2.10, ThermoScientific) software. The FA concentration was expressed in mg.g^-1^ of dry weight or mg.L^-1^ of medium, and FA composition was expressed in percentage of the total FA content (%).

Additionally, FAME’s identification was performed by GC-MS (Trace 1300 GC and ISQ7000 Single Quadrupole MS, ThermoScientific) analysis with mass scanning ranging from 40 to 600 m/z. The equipment and its settings are described in the following section.

Furthermore, the quantification of isotopic enrichment of TL was performed using GC-MS following the same chromatography protocol as GC-FID, with helium (He) as the gas carrier. The MS was operated with electron impact ionization (EI^+^) mode, emission current of 50 μA, ion source temperature of 200 °C, electron energy of 70 eV, electron lens voltage of 5 V and detector gain of 3 × 10^5^. MS analysis was performed on single ion monitoring (SIM) mode to target m/z ranges of all carbon isotopologues for 16:0 (m/z = 270–286), C23:0 (m/z = 368–391), DPA (m/z = 344–366), and DHA (m/z = 342–364) to corresponding retention times. The isotopic enrichment was estimated by a ratio of ^13^C/C_total_ according to the corresponding proportion of ^12^C carbon and ^13^C carbon from each isotopologue, as indicated in the equation below, adapted from Moreau et al. (2004) and Tejerina et al. (2023), n represents the number of ^13^C or ^12^C in the isotopologue (Moreau et al., 2004; Tejerina et al., 2023).

C 13enrichment=∑nC13 x abundance∑nC12 x abundance+∑nC13 x abundance x 100

### 2.7 Fatty acids methyl esters purification

FAME’s purification was carried out using High Performance Liquid Chromatography (HPLC, Dionex Ultimate 3000 UHPLC^+^, ThermoScientific) with a protocol adapted from Watanabe et al. (2000) and Le et al. (2007) (Watanabe et al., 2000; Le et al., 2007). Briefly, sample (100 μL, 5 g FA. L^-1^) was loaded on an Aquasil C18 column (250 × 4.6 mm, 5 μm, 100 Å, ThermoScientific). FAME separation was carried out by an isocratic mobile phase acetonitrile/H_2_O (80:20, v/v) at a flow rate of 1 mL min^−1^. The absorbance wavelength was 205 nm. DHA, DPA n-6 and 16:0 were collected separately. The purified FA were evaporated under nitrogen and solubilized in hexane for quantification through GC-FID (using external FA standard ranges) and GC-MS analysis in order to ensure purity and confirm the ^13^C-enrichment, following the protocols above.

### 2.8 Statistical analysis

Data normality was assessed using the Shapiro–Wilk test, and variance homogeneity was verified with the Levene test. ANCOVA were performed to determine significant differences (p ≤ 0.05) in cell growth and glucose consumption between culture conditions, except at T0 where no replicate measurements were available. ANOVA was used to assess differences in total fatty acid and DHA contents between culture conditions and harvesting methods. All statistical analyses were conducted in R (v4.4.1). The results were expressed as mean ± standard deviation (SD).

## 3 Results

### 3.1 Cell parameters and glucose concentration

#### 3.1.1 Aurantiochytrium mangrovei

**FIGURE 1:**
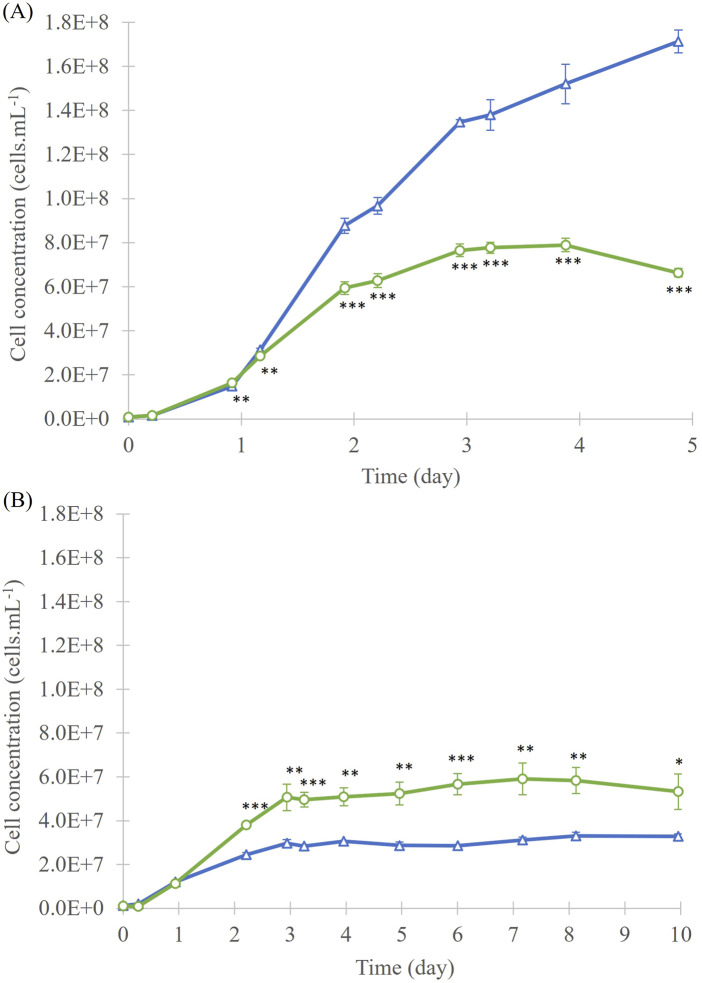
Cell concentration of *A. mangrovei* culture in 40 mL **(A)** and 200 mL **(B)** of medium, under control (blue triangles) and ^13^C-enriched (green circles) conditions, values are means ± SD (n = 3). * indicates values significantly different between culture conditions (ANCOVA; *p < 0.05, **p < 0.01, ***p < 0.001).

In the 40 mL culture experiment with *A. mangrovei*, exponential growth phase was observed between day 1 and day 3 for ^13^C-enriched and control conditions (Figure 1A). Then, it was followed by a stationary phase between day 3 and day 5 in ^13^C-enriched condition, while a slight increase between day 3 and day 5 was observed for control condition. For the “Control” condition culture, a similar latent phase was apparent, followed by an exponential phase of growth, which does not appear to have ended by the end of the 5-day culture period. At day 5, the cell concentration of the “Control” condition, at 1.7 ± 0.1 × 10^8^ cells. mL^-1^, was higher than that of the “^13^C-enriched” condition, at 6.6 ± 0.2 × 10^7^ cells. mL^-1^. Significant differences in cell growth between the two conditions were observed from day 1 (p < 0.01).

In the 200 mL culture experiment (Figure 1B), an exponential growth phase was observed from day 1 to day 3 of culture. During this phase, the average cell concentration has risen to 3.0 ± 0.2 × 10^7^ cells. mL^-1^ for the “Control” culture, and to 5.1 ± 0.6 × 10^7^ cells. mL^-1^ for the “^13^C-enriched” culture. From day 3, the stationary phase was reached and has lasted until the end of culture (day 10). Cell concentrations in “Control” cultures during the stationary phase were statistically lower than those “^13^C-enriched”. On the last day of culture, the cell density of the cultures was 3.3 ± 0.1 × 10^7^ cells. mL^-1^ for the “Control” and 5.3 ± 0.8 × 10^7^ cells. mL^-1^ for the “^13^C-enriched”. The cell concentration of the “Control” was lower after 10 days of culture than that of the “^13^C-enriched” culture. Significant differences in cell growth between the two conditions were observed from day 2 (p < 0.05). Cell growth was higher in the first experiment than in the second, and “^13^C-enriched” culture reached a higher final cell concentration in the 40 mL cultures than in the 200 mL cultures. The dry biomass collected for the “Control” condition was 297 mg, while for the “^13^C-enriched” condition it was 709 mg. This corresponded to average productivities of 49.5 mg.L^-1^. day^-1^ and 118.2 mg.L^-1^. day^-1^, respectively.

In addition to the cells’ concentration, different cellular parameters including the size, complexity and lipid content, measured by flow cytometry on the culture of *A. mangrovei* in 40 mL and 200 mL of medium, under “Control” and “^13^C-enriched” conditions are presented in Supplementary Table S1.

Regarding the size, the cells’ size remained constant in the different cultures (around 1.0 × 10^3^ A.U. for the 40 mL and 1.2 × 10^3^ A.U. (arbitrary unit) for the 200 mL), with a slight size reduction at the end of the 40 mL “Control” culture (Supplementary Table S1). In all culture conditions, cell complexity decreased at the beginning and then steadily increased while cultures were aging. The proxy of cellular lipid content in culture 40 mL remained constant at around 4.0 × 10^2^ A.U. for the “Control”, but increased in two steps for the “^13^C-enriched” culture from 3.0 × 10^2^ A.U. to 1.1 × 10^3^ A.U. In 200 mL cultures, the proxy increased from around 8.2 × 10^2^ A.U. at day 3 to 1.9 × 10^3^ A.U. at day 8, then decreased to around 1.5 × 10^3^ A.U. for both conditions.

**FIGURE 2:**
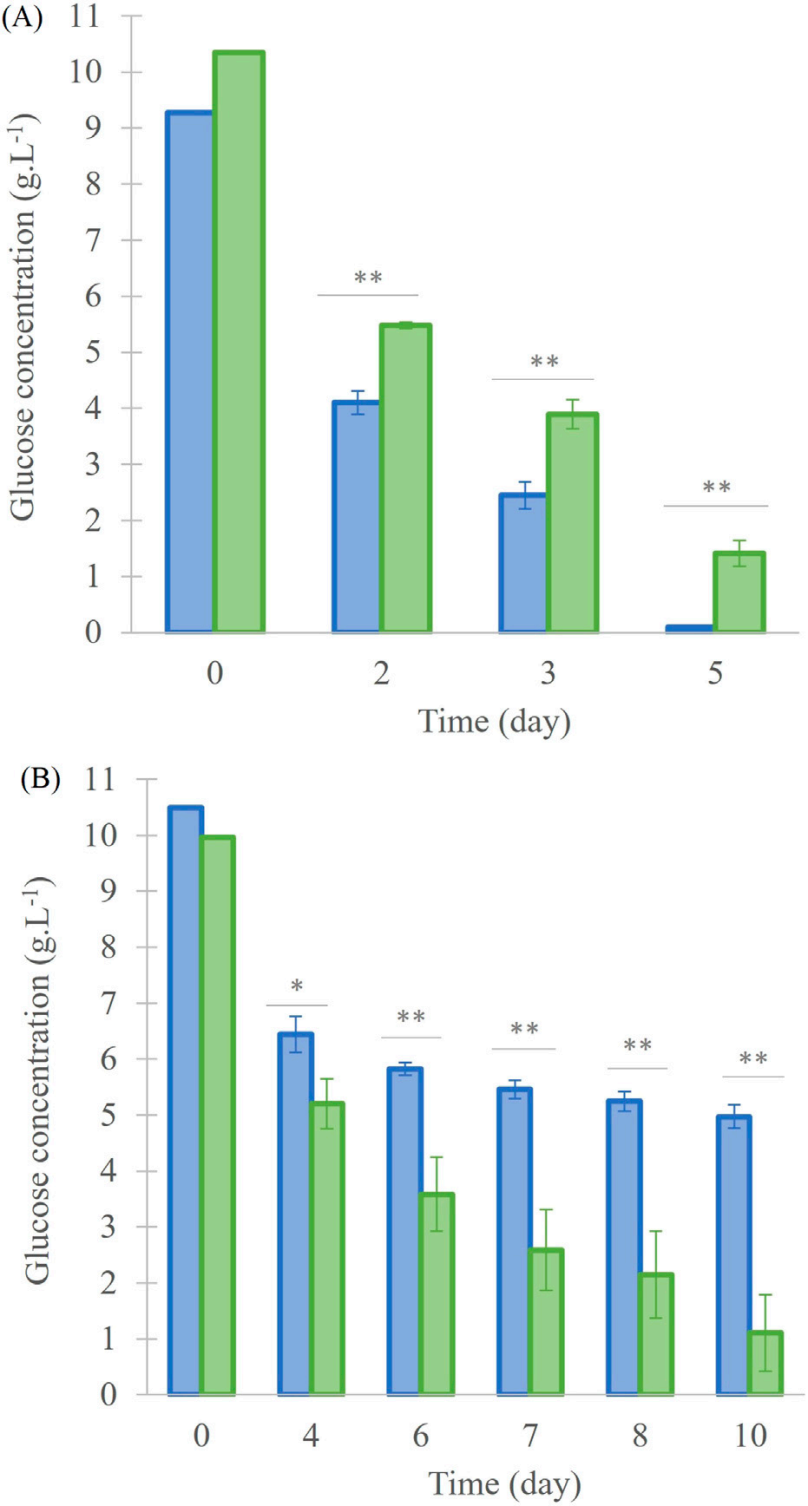
Glucose concentration in the *A. mangrovei* culture in 40 mL **(A)** and 200 mL **(B)** of medium, under control (blue) and ^13^C-enriched (green) conditions, values are means ± SD (n = 3). * indicates values significantly different between culture conditions (ANCOVA; *p < 0.05, **p < 0.01, ***p < 0.001).

Glucose consumption was measured in order to stop the culture before carbon source becomes limiting (concentration around 1 g. L^-1^ in “^13^C-enriched” condition). At the beginning, the glucose quantity for 40 mL and 200 mL cultures was at 10.0 ± 0.5 g.L^-1^ (Figure 2). For the 40 mL cultures (Figure 2A), glucose concentration decreased in “^13^C-enriched” and “Control” conditions until day 5 to 1.4 ± 0.2 g.L^-1^ and 0.10 ± 0.01 g.L^-1^, respectively, and the consumption was significantly different (p < 0.001). For 200 mL cultures (Figure 2B), the glucose concentration at day 10 was 5.0 ± 0.2 g.L^-1^ for the “Control” and 1.1 ± 0.7 g.L^-1^ for the “^13^C-enriched” culture, again with a significant difference (p < 0.001).

#### 3.1.2 Crypthecodinium cohnii

**FIGURE 3:**
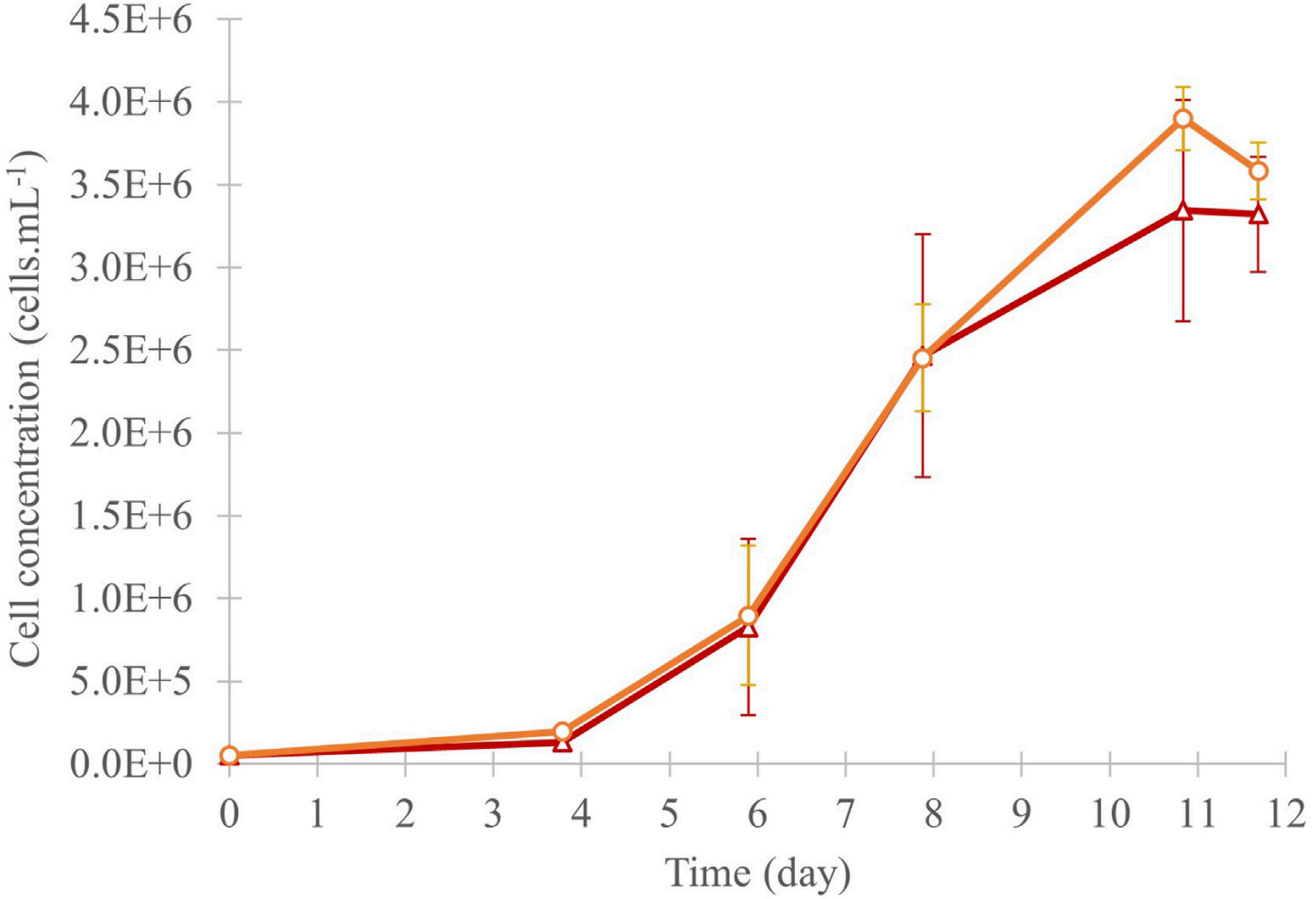
Cell concentration of the *C. cohnii* culture in 40 mL of medium, under control (red triangles) and ^13^C-enriched (orange circles) conditions, values are means ± SD (n = 3). No significant difference was observed between the conditions at day 12.

The cell growth of *C. cohnii* was statistically similar in both culture conditions, with an exponential growth from day 4 until day 11 and a declining tendency by day 12 (Figure 3). Cell numbers increased by a factor of 66 and 69 in “Control” and “^13^C-enriched” conditions, respectively, to reach a cell concentration of around 3.5 × 10^6^ cells. mL^-1^. The dry biomass collected for the “Control” condition was 182.5 mg, while for the “^13^C-enriched” condition it was 224.0 mg, corresponding, respectively, to average productivities of 126 mg.L^-1^. day^-1^ and 155 mg.L^-1^. day^-1^.

In the culture of *C. cohnii* in 40 mL of medium, under “Control” and “^13^C-enriched” conditions, cells’ size increased for the first 4 days, then decreased until day 6, to finally increase steadily until the end of the culture (Supplementary Table S2). Cell complexity, first increased over the first 4 days from 1.0 × 10^4^ A.U. to 1.5 × 10^4^ A.U., and then dropped to an average of 8.9 × 10^3^ A.U. at the end of cultures. The proxy of cellular lipid content increased between day 4 and day 8 from an average of 4.5 × 10^3^ A.U. to 7.4 × 10^3^ A.U. followed by a reduction until day 12 to 3.2 × 10^3^ A.U for both culture conditions.

**FIGURE 4:**
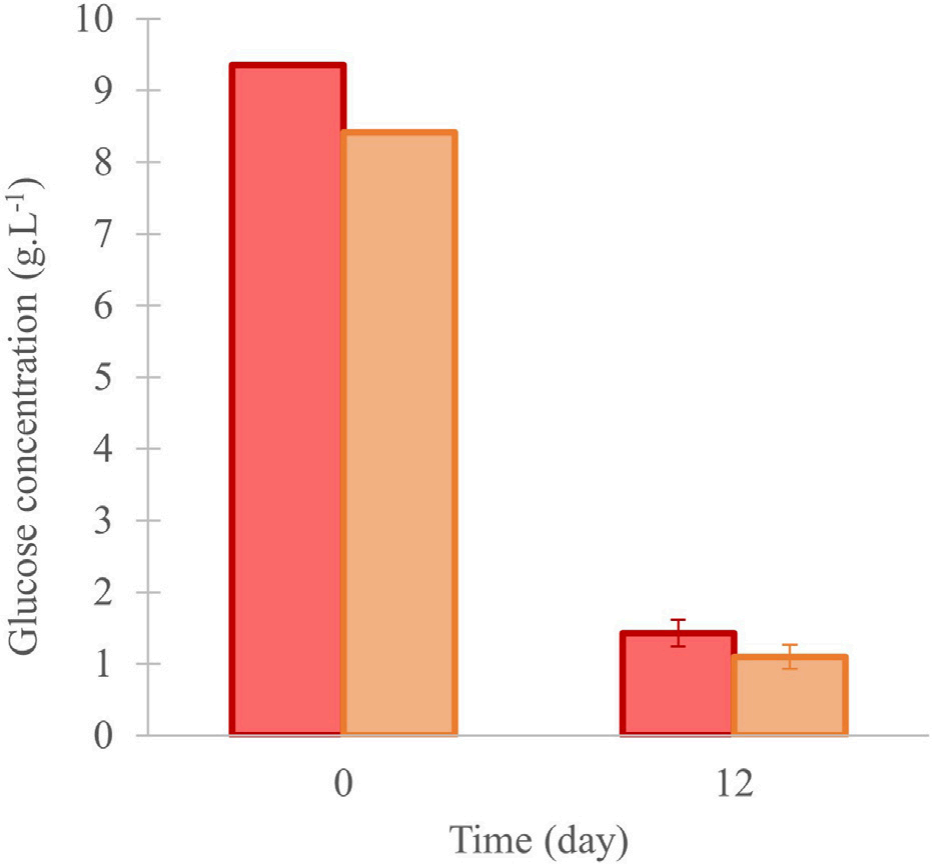
Glucose concentration in the culture of *C. cohnii* in 40 mL of medium, and under control (in red) and ^13^C-enriched conditions (in orange), values are means ± SD (n = 3). No significant difference was observed between the conditions at day 12.

The glucose’s concentration in the medium was measured at the beginning and day 12. Reductions from 9.3 g.L^-1^ to 1.4 ± 0.2 g.L^-1^ in “Control”, and from 8.4 g.L^-1^ to 1.1 ± 0.2 g.L^-1^ in “^13^C-enriched” condition were observed (Figure 4).

### 3.2 Fatty acid composition

#### 3.2.1 Aurantiochytrium mangrovei

The predominant FA in *A. mangrovei* were 16:0, DPA n-6, and DHA, independently of culture conditions including the medium volume or sampling method (Table 2). Their relative proportions to total FA were 27.4% ± 4.7% for 16:0, 9.7% ± 1.4% for DPA n-6, and 50.5% ± 4.9% for DHA. Additionally, FA present above 1% included saturated fatty acids (SFA) ranging from 14:0 to 18:0, along with 20:4n-6, 20:5n-3 and 22:5n-3. A comparative analysis between “Control” and “^13^C-enriched” cultures revealed that the relative abundance of DHA was higher in the “Control”, whereas the 16:0 was more prevalent in the “^13^C-enriched” conditions.

**TABLE 2:** Fatty acid composition of total, neutral and polar lipids (TL, NL and PL) of *A. mangrovei* in 40 mL and 200 mL of medium, under control and^13^C-enriched conditions, values are means ± (SD) (TL, n > 6, NL and PL, n = 3). The upper part presents the results in % total FA, considering FA > 1%, the lower part presents the content of DHA in mg.L^-1^ of medium (FL: filtered sample, FD: freeze-dried sample, FA: fatty acids, SFA: saturated fatty acids, MUFA: monounsaturated fatty acids, PUFA: polyunsaturated fatty acids, nd: not determined). Different letters indicate significant differences (ANOVA, p < 0.05) within total lipids.

	A40-ctrl-FL		A200-ctrl-FL		A200-ctrl-FD						A40-13C-FL		A200-13C-FL		A200-13C-FD					
% Total FA	TL		TL		TL		NL		PL		TL		TL		TL		NL		PL	
14:0	0.8	(0.0)	1.6	(0.0)	1.3	(0.1)	2.2	*(nd)*	0.6	*(nd)*	1.2	(0.1)	1.2	(0.0)	1.0	(0.0)	1.7	(0.0)	0.4	(0.0)
15:0	1.1	(0.1)	1.6	(0.1)	1.4	(0.1)	1.7	*(nd)*	1.5	*(nd)*	2.2	(0.3)	1.4	(0.0)	1.5	(0.0)	1.7	(0.0)	1.4	(0.1)
16:0	19.0	(0.3)	27.7	(0.6)	26.4	(0.7)	32.4	*(nd)*	24.9	*(nd)*	28.4	(0.3)	32.6	(0.7)	30.4	(1.1)	36.8	(1.0)	24.5	(0.6)
17:0	0.4	(0.0)	1.1	(0.1)	1.1	(0.1)	1.6	*(nd)*	0.8	*(nd)*	1.1	(0.1)	1.1	(0.0)	1.1	(0.0)	1.5	(0.1)	0.7	(0.0)
18:0	0.1	(0.0)	0.9	(0.0)	0.9	(0.0)	2.2	*(nd)*	0.6	*(nd)*	1.1	(0.0)	1.6	(0.1)	1.4	(0.1)	2.9	(0.2)	0.4	(0.1)
20:4n-6	1.0	(0.1)	1.7	(0.0)	1.8	(0.0)	1.5	*(nd)*	2.0	*(nd)*	1.1	(0.0)	1.7	(0.0)	1.9	(0.0)	1.6	(0.0)	2.2	(0.1)
20:5n-3	3.8	(0.1)	2.5	(0.1)	2.9	(0.1)	2.3	*(nd)*	3.6	*(nd)*	2.6	(0.1)	2.0	(0.1)	2.2	(0.1)	1.7	(0.1)	2.8	(0.1)
22:5n-6	12.2	(0.1)	8.4	(0.1)	8.7	(0.1)	6.2	*(nd)*	9.5	*(nd)*	9.0	(0.0)	9.8	(0.1)	10.2	(0.1)	7.6	(0.0)	12.3	(0.2)
22:5n-3	1.0	(0.0)	1.3	(0.1)	1.6	(0.1)	1.9	*(nd)*	1.5	*(nd)*	0.8	(0.0)	0.9	(0.0)	1.1	(0.1)	1.5	(0.2)	1.0	(0.1)
22:6n-3	59.0	(0.5)	50.6	(0.2)	51.3	(0.5)	43.8	*(nd)*	51.4	*(nd)*	50.7	(0.2)	45.1	(0.7)	46.3	(1.1)	38.5	(0.9)	50.9	(0.6)
Other	1.7	(0.2)	2.6	(0.0)	2.7	(0.0)	4.1	*(nd)*	3.6	*(nd)*	1.8	(0.1)	2.5	(0.1)	3.0	(0.1)	4.7	(0.1)	3.3	(0.2)
Total FA	100	(0.0)	100	(0.0)	100	(0.0)	100	*(nd)*	100	*(nd)*	100	(0.0)	100	(0.0)	100	(0.0)	100	(0.0)	100	(0.0)
Total SFA	21.5	(0.4)	33.2	(0.6)	31.3	(0.8)	40.4	*(nd)*	28.6	*(nd)*	34.2	(0.3)	38.1	(0.7)	35.6	(1.2)	44.9	(1.2)	27.7	(0.6)
Total MUFA	0.3	(0.0)	0.5	(0.0)	0.8	(0.0)	1.3	*(nd)*	1.5	*(nd)*	0.3	(0.1)	0.4	(0.0)	0.8	(0.0)	1.7	(0.2)	1.3	(0.2)
Total PUFA	78.2	(0.4)	66.4	(0.6)	67.9	(0.8)	58.3	*(nd)*	69.9	*(nd)*	65.6	(0.3)	61.5	(0.7)	63.6	(1.3)	53.4	(1.2)	71.0	(0.6)
Total FA (mg.L-1)	TL		TL		TL		NL		PL		TL		TL		TL		NL		PL	
22:6n-3	107.2^a^	(9.4)	45.5^b^	(1.4)	32.8^b^	(2.2)	8.3	*(nd)*	11.8	*(nd)*	104.1^ac^	(2.6)	84.8^cd^	(10.1)	105.4^d^	(13.7)	22.1	(2.2)	29.4	(2.6)
Total	181.6^a^	(17.3)	90.0 ^b^	(2.5)	64.1 ^b^	(4.9)	19.1	*(nd)*	22.9	*(nd)*	205.2 ^ac^	(5.8)	187.1 ^cd^	(19.2)	227.6^d^	(24.7)	57.3	(4.7)	57.7	(4.6)

To compare the different harvesting methods and select the optimal one, the quantities of FA and DHA were calculated in mg FA·L^-1^ of medium. In 40 mL cultures, the total FA and DHA content were statistically comparable between conditions, despite lower cell growth under “^13^C-enriched” conditions. However, in 200 mL cultures, the “^13^C-enriched” samples exhibited a higher total FA and DHA content (p < 0.001), particularly for A200-13C-FD. When comparing the two harvesting methods for the “Control” and “^13^C-enriched” cultures, no statistically significant trend favored one method over the other. The NL and PL contents were quantified only for the FD samples based on FA contents, representing 47.6% ± 3.1% and 52.4% ± 3.1% of the TL, respectively. Focusing on the total DHA content, 42.2% ± 1.0% was found in the NL and 57.8% ± 3.1% in the PL.

#### 3.2.2 Crypthecodinium cohnii

*C. cohnii* primarily contained 12:0, 14:0, 16:0 and DHA, regardless of the culture conditions or the sampling method (Table 3). The proportions of 12:0, 14:0 and DHA varied depending on the harvesting method. For 12:0, the proportions were 10.9% ± 2.1% of the total FA in C40-FL and 16.9% ± 0.5% in C40-FD. For 14:0, the proportions were 22.5% ± 0.6% of the total FA in C40-FL and 32.2% ± 0.5% in C40-FD. For DHA, the proportions were 36.5% ± 1.9% and 22.6% ± 0.1% for C40-FL and C40-FD, respectively. Lastly, the proportion of 16:0 remained consistent at 15.9% ± 0.7% of total FA under both FL and FD conditions. The two culture conditions exhibited statistically similar FA and DHA contents. When comparing the FA contents in mg FA·L^-1^, the FA contents were statistically higher in the freeze-dried samples (p < 0.001). The proportions of NL and PL were determined only for the FD samples based on FA contents, with 82.4% ± 0.3% NL, containing 57.5% ± 1.0% of the total DHA, and 17.6% ± 0.3% PL, containing 42.5% ± 1.0% of the total DHA.

**TABLE 3:** Fatty acid composition of total, neutral and polar lipids (TL, NL and PL) of *C. cohnii* in 40 mL of medium, under control and^13^C enriched conditions, values are means ± (SD) (TL, n > 6, NL and PL, n = 3). The upper part presents the results in % total FA, considering FA > 1%, the lower part presents the content of DHA in mg.L^-1^ of medium (FL: filtered sample, FD: freeze-dried sample, FA: fatty acids, SFA: saturated fatty acids, MUFA: monounsaturated fatty acids, PUFA: polyunsaturated fatty acids, nd: not determined). Different letters indicate significant differences (ANOVA, p < 0.05) within total lipids.

	C40-ctrl-FL		C40-ctrl-FD						C40-13C-FL		C40-13C-FD					
% Total FA	TL		TL		NL		PL		TL		TL		NL		PL	
12:0	12.4	(0.2)	17.2	(0.2)	20.5	(nd)	1.3	(nd)	9.4	(0.3)	16.5	(0.4)	20.5	(0.6)	1.0	(0.2)
14:0	22.9	(0.2)	31.8	(0.7)	40.0	(nd)	12.8	(nd)	22.0	(0.1)	32.5	(0.3)	40.0	(0.5)	12.7	(0.4)
16:0	15.0	(0.0)	15.9	(0.1)	15.0	(nd)	19.6	(nd)	16.7	(0.2)	16.1	(0.1)	15.4	(0.2)	21.0	(0.5)
18:0	1.3	(0.0)	2.1	(0.0)	2.2	(nd)	2.3	(nd)	1.3	(0.1)	2.1	(0.0)	2.3	(0.1)	3.0	(0.2)
18:1n-9	8.2	(0.1)	5.6	(0.1)	3.6	(nd)	12.1	(nd)	8.1	(0.2)	5.0	(0.1)	3.3	(0.1)	11.5	(0.8)
22:6n-3	35.1	(0.2)	22.4	(0.7)	13.3	(nd)	46.6	(nd)	37.8	(0.1)	22.7	(0.5)	13.3	(0.8)	45.4	(1.3)
28:8n-3	2.0	(0.1)	1.3	(0.0)	0.7	(nd)	1.9	(nd)	2.1	(0.1)	1.5	(0.0)	0.8	(0.1)	2.0	(0.2)
Total FA	100	(0.0)	100	(0.0)	100	(nd)	100	(nd)	100	(0.0)	100	(0.0)	100	(0.0)	100	(0.0)
Total SFA	52.7	(0.2)	68.3	(1.0)	79.2	(nd)	37.1	(nd)	50.3	(0.1)	68.6	(0.6)	79.6	(1.2)	38.8	(0.7)
Total MUFA	9.5	(0.0)	7.5	(0.2)	6.3	(nd)	13.2	(nd)	9.3	(0.2)	6.8	(0.1)	5.9	(0.4)	12.5	(0.8)
Total PUFA	37.8	(0.2)	24.2	(0.8)	14.5	(nd)	49.7	(nd)	40.5	(0.2)	24.6	(0.6)	14.5	(0.9)	48.7	(1.1)
Other	2.8	(0.1)	3.6	(0.1)	4.5	(nd)	3.3	(nd)	2.6	(0.0)	3.5	(0.1)	4.3	(0.4)	3.3	(0.1)
FA (mg.L-1)	TL		TL		NL		PL		TL		TL		NL		PL	
22:6n-3	51.8^ac^	(4.1)	45.1^bc^	(0.4)	11.0	(nd)	8.4	(nd)	56.3^ad^	(1.5)	50.7^bd^	(3.4)	12.3	(1.1)	8.8	(0.4)
Total FA	147.5^a^	(12.2)	201.2^b^	(6.2)	82.6	(nd)	18.0	(nd)	149.0^a^	(4.2)	223.6^b^	(9.6)	92.2	(2.4)	19.4	(1.4)

### 3.3 HPLC purification of ^13^C-DHA

**FIGURE 5:**
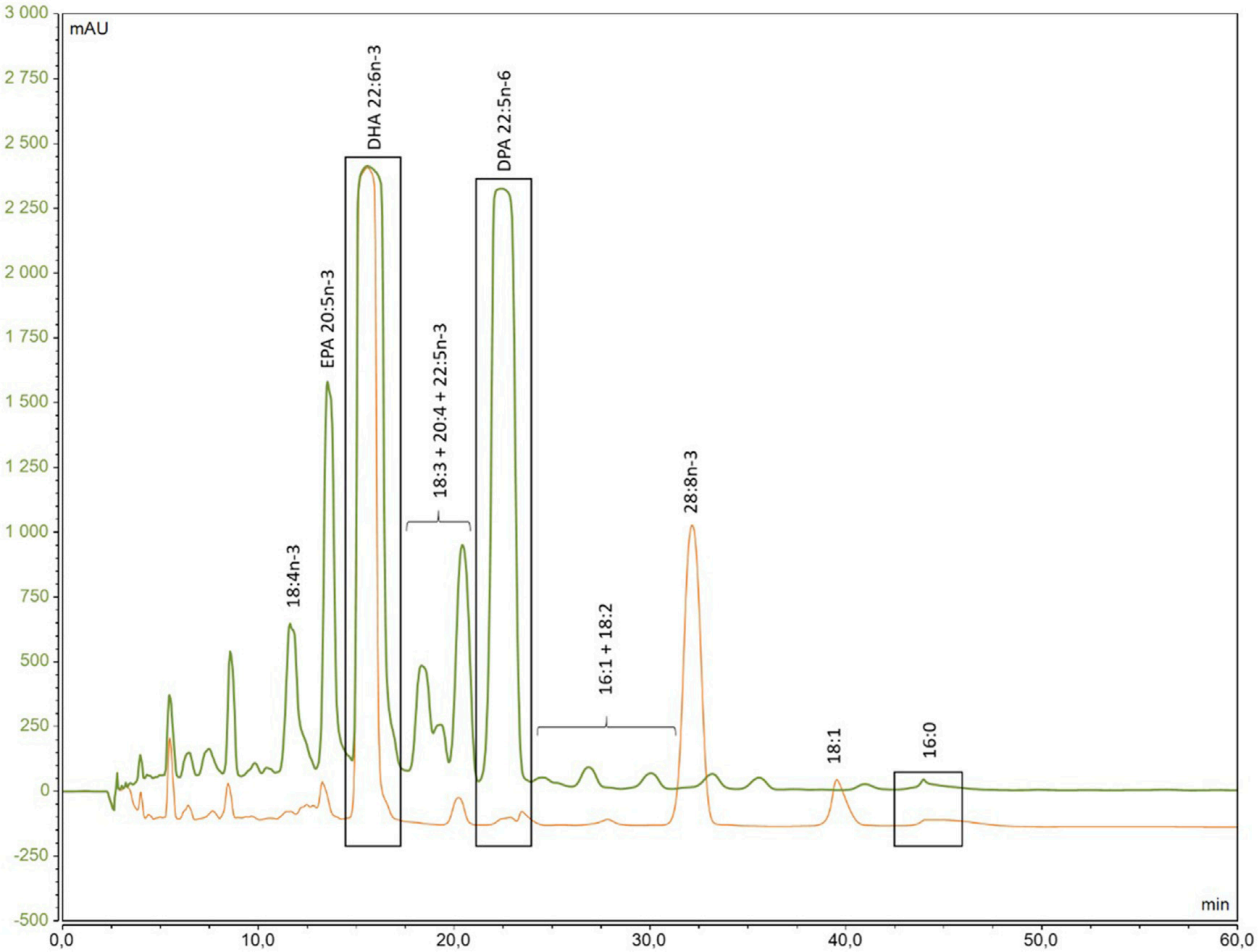
Chromatogram of the separation of ^13^C-enriched fatty acids methyl ester from *A. mangrovei* (green) and *C. cohnii* (orange) by HPLC.

Following FAME production, in order to purify DHA from other FA, HPLC purification was conducted as demonstrated in the chromatogram of the two species (Figure 5), where their distinct retention times were clearly observed. Although DHA was the primary target, all peaks were collected, as well as the intermediate fractions between these FA to confirm the identification.

### 3.4 Mass spectra and ^13^C-enrichment of DHA

#### 3.4.1 Aurantiochytrium mangrovei

**FIGURE 6:**
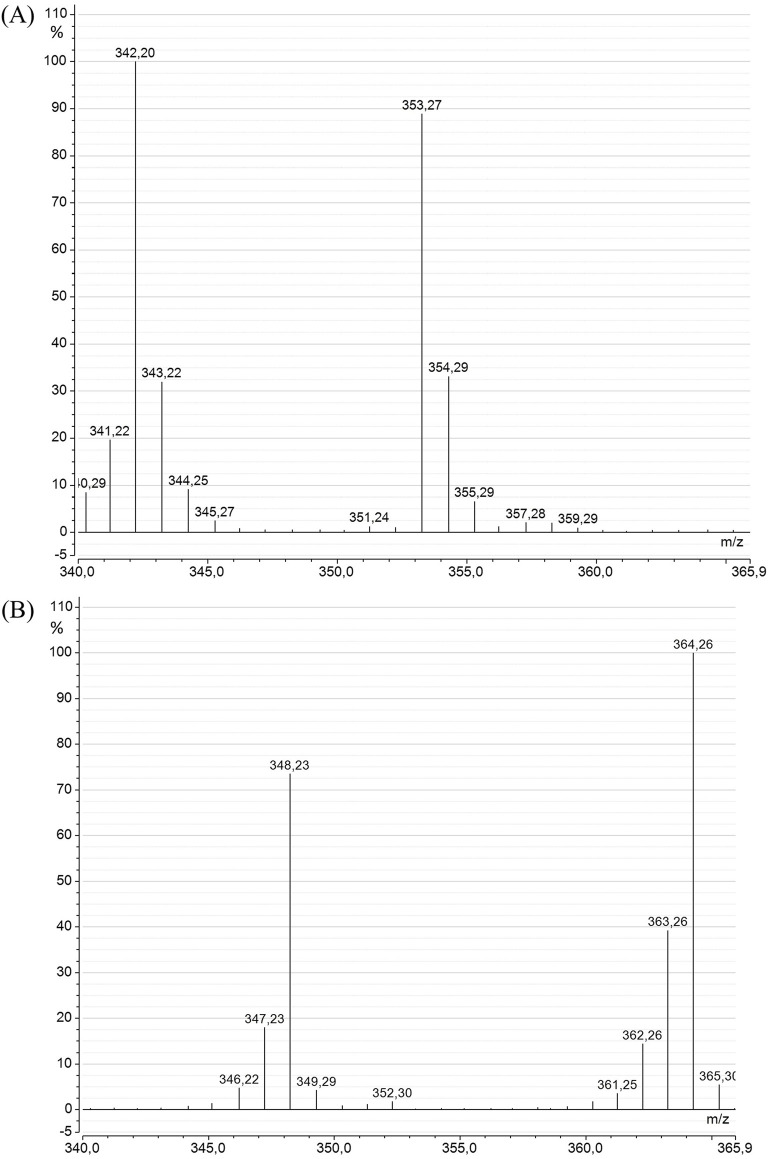
Mass spectra of DHA methyl ester from *A. mangrovei* in the SIM mode (m/z = 340–366), for the “Control” **(A)** and the “^13^C-enriched” **(B)** conditions.

The SIM-mode mass spectra of DHA (Figure 6) from *A. mangrovei* cultures revealed a drastic modification between the “Control” and “^13^C-enriched” conditions. Specifically, the spectrum of DHA in methyl ester form in control condition displayed a molecular ion at m/z = 342, accompanied by several small fragments likely reflecting background noise or the natural isotopic abundance of atoms within the molecule. Previous analyses of this model using GC-c-IRMS estimated the natural enrichment in ^13^C at approximately 1.1% (unpublished data). Under “^13^C-enriched” condition, the mass of the molecular ion shifted to m/z = 364, corresponding to ^13^C_22_-DHA and confirming that all carbons are ^13^C-labeled. Another notable fragment was observed at m/z = 348, which appeared to result from the loss of a^13^C-methyl group from the DHA acyl chain, [M-16]^+^. Consequently, ^13^C-enrichment was calculated based on the fragments between m/z 353–364 to eliminate the contribution of the lower fragment [M–16]^+^, which was also detected in the control at [M–15]^+^, as the carbon was ^12^C. Following HPLC purification, the analysis indicated a DHA enrichment of 96.7% ± 0.4%.

#### 3.4.2 Crypthecodinium cohnii

**FIGURE 7:**
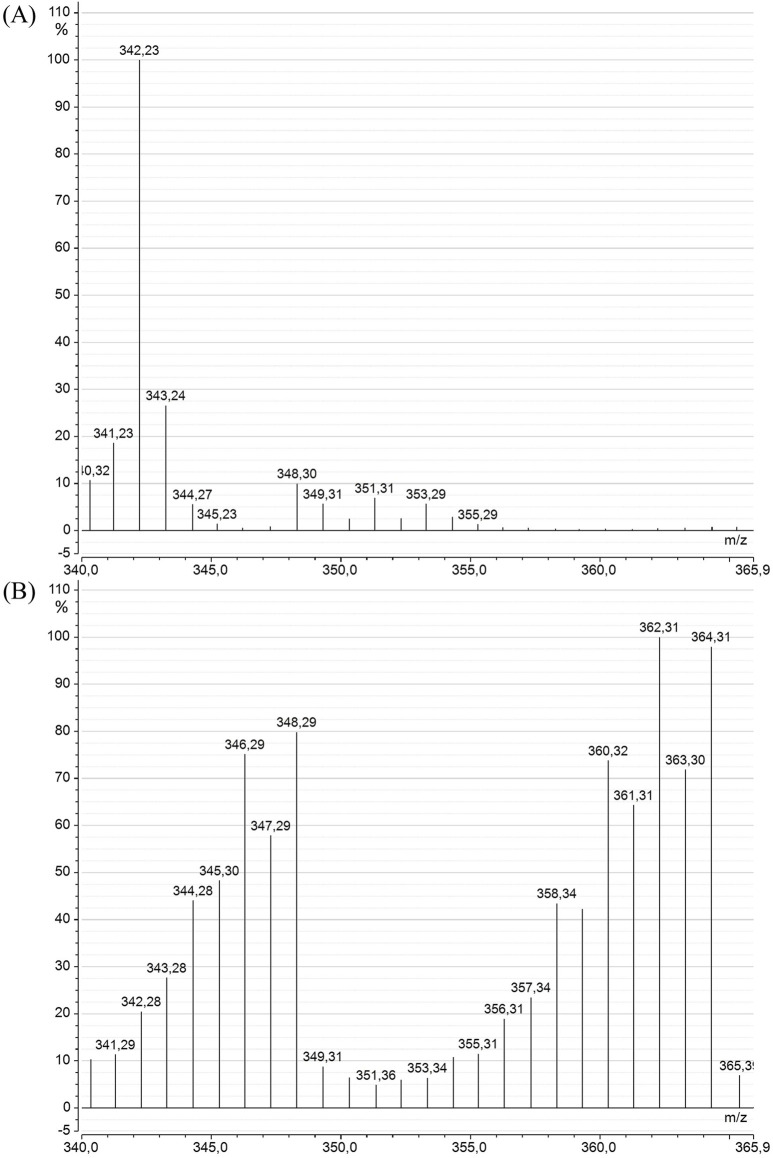
Mass spectra of DHA methyl ester from *C. cohnii* in the SIM mode (m/z = 340–366), for the “Control” **(A)** and the “^13^C-enriched” **(B)** conditions.

The SIM-mode mass spectra of DHA (Figure 7) from *C. cohnii* cultures also revealed a significant alteration in the DHA mass spectrum between the “Control” and “^13^C-enriched” conditions. The spectrum of the control DHA was similar to that of *A. mangrovei* DHA, except that there was no interference from other major fragments within the measurement range, and the molecular ion was clearly observed at m/z = 342. In earlier work, GC-c-IRMS measurements indicated a natural ^13^C enrichment of 1.1% (data not shown). The DHA spectrum under the “^13^C-enriched” condition differed not only from the control but also from the ^13^C-enriched DHA of *A. mangrovei*, suggesting a lower level of enrichment for *C. cohnii* samples. This was supported by the enrichment calculation, which yielded values of 86.3% ± 1.6% after HPLC purification.

### 3.5 Quantification of purified ^13^C-DHA

**FIGURE 8:**
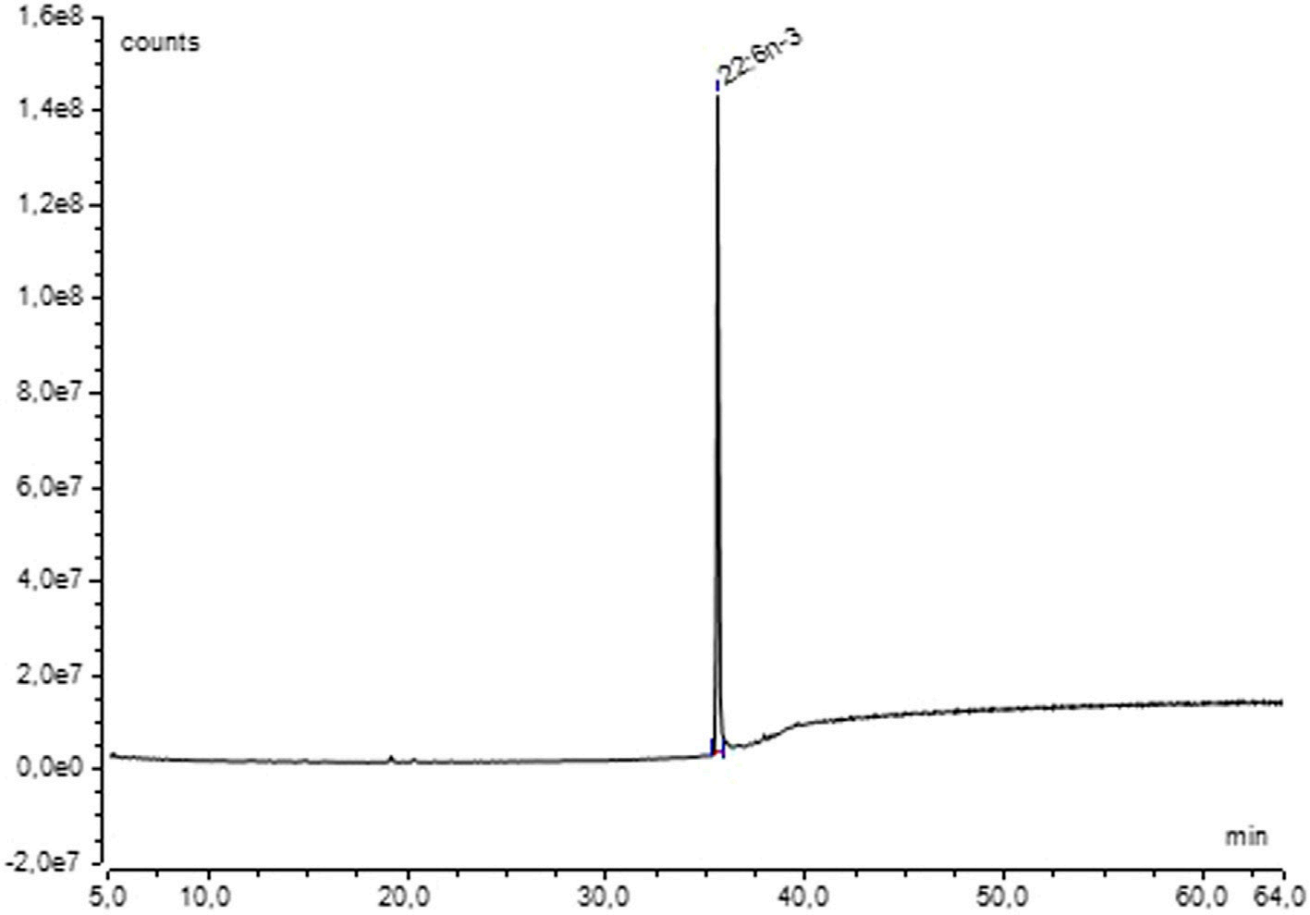
Chromatogram of ^13^C-DHA methyl ester from *A. mangrovei* in the SIM mode (m/z = 340–366) after HPLC purification.

For quantification, all ^13^C-enriched samples were pooled by species. GC-FID analysis of the purified ^13^C-DHA of *A. mangrovei* indicated that the purified amount was 28 ± 6 mg, with only half of the lipid extracts. Therefore, the total amount of ^13^C-DHA that could have been obtained was approximately 56 mg. Based on the DHA content determined by GC-FID analysis, the total collected culture medium (613.5 mL) contained approximately 59.4 mg of DHA, corresponding to a purification yield of 94.3%. In the case of *C. cohnii*, the purified quantity was 2 mg with only half of the lipid extracts, thus, a total of 4 mg of ^13^C-DHA from all samples. Based on GC-FID quantification, the total collected culture medium (105 mL) contained about 5.7 mg of DHA, yielding a purification efficiency of 70.2%. The chromatogram obtained by GC-MS (Figure 8) demonstrated that DHA purification was complete for both species, as it was the only peak observed (retention time of 35.5 min).

## 4 Discussion

### 4.1 Culture growth and lipid content

*A. mangrovei* cell growth in 40 mL cultures was significantly higher in the “Control” condition compared to the “^13^C-enriched” condition associated with a higher glucose consumption, reaching 99.0% and 86.4%, respectively. Moreover, in the “Control” condition, the stationary phase had not been reached by day 5, indicating that the cells remained in the exponential growth phase when the culture was halted. This was further supported by the smaller size and lower complexity of the cells, characteristic of cells actively undergoing division. In contrast, cells tend to increase in size and accumulate lipids during the stationary phase (Opute, 1974). Since the “^13^C-enriched” condition reached the stationary phase by day 3, the cells were likely lipid-loaded, as confirmed by lipid content analysis using the “Bodipy” marker. The only difference between the culture media was the type of glucose used (^12^C or ^13^C), suggesting that the presence of ^13^C-glucose may have influenced cell growth. Previous studies have shown that high levels of ^13^C-enrichment can induce changes in molecular configuration or conformation (Ritchie et al., 2017). Such conformational changes could arise from the incorporation of ^13^C-glucose into biomolecules, such as proteins, potentially altering their function and affecting cellular metabolism.

In the 200 mL cultures, cell growth in “^13^C-enriched” condition was significantly higher than in the “Control” condition starting from day 2 during the exponential phase. Notably, the “Control” cultures exhibited reduced growth, with a 6-fold decrease by day 5 compared to the 40 mL cultures, accompanied by a 2.2-fold lower glucose consumption (44.5%). This difference may be attributed to the physiological state of the cells at the inoculation time into the 200 mL medium, as they were in the exponential growth phase, which may explain their impaired ability to restart a subsequent exponential phase. In contrast, the “^13^C-enriched” cultures, which were inoculated during the stationary phase, maintained a more stable growth pattern. This was likely due to their higher cell complexity and sufficient energy reserves, allowing them to successfully reinitiate exponential cell division. Both cultures reached the stationary phase by day 3, at which point cells began accumulating lipids. Lipid content monitoring revealed a similar increase in lipid accumulation in both conditions from days 4–8. After this period, a decline in lipid content was observed, likely due to the deterioration of the cells’ physiological state and the depletion of glucose, the primary carbon source, induced the cell to use the carbon stored in lipid reserves to sustain survival.

During *C. cohnii* cultures, both conditions exhibited similar trends and results. Over the 12-day culture period, cell concentrations increased more than 65-fold, accompanied by glucose consumption of 85.8% ± 1.6%. Glucose and yeast extract have previously been identified as an effective source of organic carbon and nitrogen for promoting cell development in this species (Cui et al., 2018; Mendes et al., 2009). Cell size fluctuated depending on the growth phase, with smaller cells observed during cell divisions and larger cells during the stationary phase. Cell complexity increased during the lag phase but dropped below its initial value as growth progressed. Harvesting was performed when the stationary phase was being established, though lipid levels had already begun to decline by day 8, as likely as a response to glucose depletion.

### 4.2 Fatty acid composition

The three major FA found in *A. mangrovei* cultures were 16:0, DPA n-6, and DHA, with respective contents of 27.4% ± 4.7%, 9.7% ± 1.4%, and 50.5% ± 4.9% of total FA. These predominant FA were consistent with those reported by Soudant et al. (2022), although the contents were slightly different: 34.3% for 16:0, 12.3% for DPA n-6, and 40.5% for DHA (Soudant et al., 2022). Therefore, the culture conditions of the current study favored DHA’s production. It was observed that DHA content was higher in the “Control” condition, while the proportions of 16:0 were higher in the “^13^C-enriched” condition. This could be due to the impact of ^13^C atoms on DHA synthesis and their possible effect on the enzymes involved. The FA content in the 40 mL culture was similar between conditions, but in the 200 mL culture, it was higher under the “^13^C-enriched” condition, likely due to the physiological state of the cells at the time of inoculation and the associated lower glucose consumption.

Furthermore, despite significant differences in cell growth, the amount of DHA produced in the 40 mL cultures was comparable. This can be explained by the fact that the “Control” cultures exhibited higher cell growth but had smaller sizes and lower lipid content. Consequently, this interplay between growth rate, cell size, and lipid accumulation resulted in similar DHA production between both conditions.

In the 200 mL cultures, both extraction methods yielded comparable FA percentages across conditions. However, filtration proved difficult at high cell densities, making freeze-drying more suitable for large-scale extractions. Filtered samples showed differences in FA and DHA contents likely due to variations in cell growth, whereas freeze-dried samples showed reduced differences, as values were normalized to biomass dry weight. By the end of cultivation, lipid contents were similar between conditions, as confirmed by BODIPY staining. The proportions of neutral and polar lipids with high DHA content were balanced, which may indicate a non-stressful culture. Although cell growth remained stable, the cells had not yet initiated energy storage. Moreover, cell concentration in the 200 mL culture remained low compared to the 40 mL control, despite the presence of glucose in the medium, suggesting that another limiting factor may have constrained further growth.

In *C. cohnii* cultures, the majors FA were 12:0, 14:0, 16:0 and DHA, accounting for 13.9% ± 3.7%, 27.3% ± 5.6%, 15.9% ± 0.7% and 29.5% ± 8.1% of the total FA, respectively. Other studies have reported similar DHA levels, such as Mendes et al. (2009), who reported approximately 30% DHA with other PUFA below 1%, and Cui et al. (2018), where DHA represented around 42% (Cui et al., 2018; Mendes et al., 2009). DPA n-6 was not detected in *C. cohnii*, which is consistent with previous study and may facilitate the purification of DHA (Cui et al., 2018). In *C. cohnii*, the concentrations of neutral and polar lipids were unbalanced, with a high proportion of NL. This is consistent with the physiological state of the cells at the time of harvest, stationary phase, during which cell division had ceased or slowed down, and cells accumulated neutral lipids, as energy compounds.

### 4.3 DHA enrichment and purification

The enrichment of DHA was analyzed using GC-MS, revealing a shift of 22 m/z units, from a molecular ion at m/z = 342 for the “Control” DHA to m/z = 364 for the “^13^C-enriched” DHA. The mass spectra indicated that the predominant isotopologues of ^13^C-DHA were ^13^C_22_H_32_O_2_, ^13^C_21_^12^CH_32_O_2_, and ^13^C_20_^12^C_2_H_32_O_2_. Following the effective purification of DHA using HPLC, ^13^C-enrichment was 96.7% ± 0.4% for *A. mangrovei* and was 86.3% ± 1.6% for *C. cohnii*. The lower enrichment of DHA synthesized by *C. cohnii* was possibly due to the presence of ^12^C in the yeast extract, although glucose was the primary source of organic carbon. Previous studies on heterotrophic microorganisms have also demonstrated similar high levels of enrichment, notably *Schizochytrium sp.*, which synthesized ^13^C-DHA with 97% enrichment (Watanabe et al., 2000), *Hyalochlorella marina*, which produced ^13^C-DHA with over 90% enrichment (Le et al., 2007), and *C. cohnii*, which produced ^13^C-DHA enriched to 96.8% (Song et al., 2020), with culture conditions similar to those used here for *A. mangrovei*.

When considering the average biomass productivities under ^13^C-enriched conditions, *C. cohnii* was about 1.3-fold more productive. Nevertheless, its total fatty acid and DHA contents were lower at the end of the culture. Thus, *A. mangrovei* appeared to be the more promising organism for further work, as it achieved a greater DHA enrichment, likely influenced by the specific composition of its culture medium, which is not compatible with *C. cohnii*.

The production of ^13^C-enriched DHA by *A. mangrovei* was 98 ± 12 mg.L^-1^ of culture, and by *C. cohnii* was 54 ± 4 mg.L^-1^ of culture. The DHA production by *A. mangrovei* was similar to that reported by Watanabe et al. (2000), with the Thraustochytrid strain, *Schizochytrium sp*., which not exceeding 100 mg.L^-1^ of DHA produced when using glucose, with comparable cell growth (Watanabe et al., 2000). In contrast, their cultivation with ^13^C-sodium acetate was more promising, reaching up to 700 mg.L^-1^ within 100 h before a decline in concentration. The DHA yield relative to the glucose consumed was approximately 11 mg DHA. g^-1^ glucose and 7 mg DHA. g^-1^ glucose for *A. mangrovei* and *C. cohnii*, respectively. Both GC-MS and GC-FID analyses confirmed the absence of other FA, indicating that the final enriched DHA had a purity near 100%, consistent with results reported in the literature (Watanabe et al., 2000). The HPLC purification yield were 94.3% for *A. mangrovei* and 70.2% for *C. cohnii*. The lower value obtained for *C. cohnii* likely resulted from the small DHA amount produced in its culture, which may not be representative of the true purification efficiency. While the high yield achieved for *A. mangrovei* demonstrate the robustness of the process, although further methodological improvements are required for successful scale-up. The purified yield appeared low considering the time required for purification: 1-h HPLC run purified 100 µL of FAME solution at 5 g FA. L^-1^. To enhance the yield, this time-consuming step could potentially be substituted with Flash chromatography, enabling the purification of larger quantities per run. Nevertheless, additional time may be needed to optimize the procedure.

In this study, DHA was purified in its methyl ester form; however, it was also demonstrated that both neutral and polar lipids contained DHA. This opens the possibility of purifying specific lipid classes to produce ^13^C-enriched DHA lipids, such as phospholipids and TAG, for use in fluxomic analyses. These lipid classes could be isolated using a liquid chromatography, and the purified fractions subsequently characterized by LC-MS/MS. Moreover, by adjusting the harvesting time of the microorganisms, it would be possible to favor either neutral or polar lipids.

## 5 Conclusion

This study demonstrated that the culture of *A. mangrovei* and *C. cohnii* strains enabled the production of lipids enriched in ^13^C-DHA and validated the HPLC purification method. Future work should focus on developing Flash chromatography purification methods to increase yields and reduce processing time. Additionally, purifying ^13^C-DHA enriched phospholipids and TAG will facilitate ^13^C based MFA using Liquid chromatography coupled with tandem mass spectrometry (LC-MS/MS) and GC-c-IRMS. These advancements could provide valuable insights for biotechnological applications and metabolic studies.

## Data Availability

The original contributions presented in the study are included in the article/Supplementary Material, further inquiries can be directed to the corresponding author.
