# Supplementary material for: Production of 13C-labeled docosahexaenoic acid from heterotrophic marine microorganisms Aurantiochytrium mangrovei and Crypthecodinium cohnii enabling fluxomic applications
Source: Front Bioeng Biotechnol. 2025 Nov 19;13:1690863. doi: 10.3389/fbioe.2025.1690863 (PMC12672524; doi:10.3389/fbioe.2025.1690863)
Supplement: Supplementary file 1 [file Table1.docx]

**Table S1** Cellular parameters (size, complexity, lipid content) as measured by flow cytometry on the *A. mangrovei* culture in 40 mL and 200 mL of medium, under control and ^13^C‑enriched conditions, values are means ± (SD) (n = 3) (A.U.: arbitrary units, n.d.: not determined)

| **Time (day)** | **Size (A.U.)** | | **Complexity (A.U.)** | | **Lipid Content (A.U.)** | |  |
| --- | --- | --- | --- | --- | --- | --- | --- |
| **A40-ctrl** | | | | | | | |
| 0.0 | 1104 | (0) | 246 | (0) | n.d. | n.d. |  |
| 0.2 | 940 | (34) | 160 | (2) | n.d. | n.d. |  |
| 0.9 | 1083 | (20) | 124 | (1) | 311 | (5) |  |
| 1.2 | 1044 | (8) | 116 | (1) | n.d. | n.d. |  |
| 1.9 | 885 | (11) | 121 | (1) | 371 | (8) |  |
| 2.2 | 860 | (18) | 116 | (2) | 503 | (20) |  |
| 2.9 | 893 | (11) | 128 | (1) | 431 | (14) |  |
| 3.2 | 888 | (1) | 128 | (2) | n.d. | n.d. |  |
| 3.9 | 972 | (6) | 141 | (1) | 395 | (6) |  |
| 4.9 | 729 | (11) | 142 | (3) | 400 | (35) |  |
| **A40-13C** | | | | | | | |
| 0.0 | 1085 | (0) | 245 | (0) | n.d. | n.d. |  |
| 0.2 | 957 | (9) | 144 | (2) | n.d. | n.d. |  |
| 0.9 | 1038 | (9) | 126 | (2) | 297 | (2) |  |
| 1.2 | 1126 | (14) | 136 | (1) | n.d. | n.d. |  |
| 1.9 | 1154 | (6) | 225 | (5) | 570 | (20) |  |
| 2.2 | 1102 | (23) | 226 | (2) | 812 | (20) |  |
| 2.9 | 1063 | (23) | 286 | (6) | 742 | (36) |  |
| 3.2 | 1040 | (21) | 295 | (10) | n.d. | n.d. |  |
| 3.9 | 1080 | (28) | 330 | (10) | 842 | (28) |  |
| 4.9 | 1099 | (24) | 307 | (10) | 1134 | (100) |  |
| **A200-ctrl** | | | | | | | |
| 0.0 | 992 | (16) | 126 | (2) | n.d. | n.d. |  |
| 0.3 | 1111 | (12) | 116 | (2) | n.d. | n.d. |  |
| 0.9 | 1264 | (9) | 165 | (6) | n.d. | n.d. |  |
| 2.2 | 1306 | (19) | 222 | (6) | n.d. | n.d. |  |
| 2.9 | 1149 | (29) | 219 | (9) | 889 | (26) |  |
| 3.3 | 1184 | (19) | 228 | (5) | n.d. | n.d. |  |
| 4.0 | 1193 | (2) | 250 | (10) | 1121 | (51) |  |
| 5.0 | 1153 | (44) | 271 | (6) | 1231 | (31) |  |
| 6.0 | 1100 | (11) | 304 | (13) | 1283 | (16) |  |
| 7.2 | 1092 | (18) | 316 | (10) | 1590 | (26) |  |
| 8.1 | 1084 | (18) | 331 | (6) | 1853 | (76) |  |
| 10.0 | 1041 | (60) | 349 | (14) | 1334 | (78) |  |
| **A200-13C** | | | | | | | |
| 0.0 | 1112 | (30) | 325 | (4) | n.d. | n.d. |  |
| 0.3 | 1348 | (15) | 276 | (7) | n.d. | n.d. |  |
| 0.9 | 1134 | (21) | 129 | (1) | n.d. | n.d. |  |
| 2.2 | 1138 | (14) | 203 | (9) | n.d. | n.d. |  |
| 2.9 | 1068 | (27) | 234 | (13) | 746 | (41) |  |
| 3.3 | 1121 | (16) | 241 | (15) | n.d. | n.d. |  |
| 4.0 | 1190 | (38) | 288 | (17) | 1089 | (47) |  |
| 5.0 | 1162 | (74) | 307 | (3) | 1241 | (69) |  |
| 6.0 | 1159 | (60) | 338 | (14) | 1329 | (32) |  |
| 7.2 | 1177 | (34) | 348 | (8) | 1656 | (148) |  |
| 8.1 | 1124 | (52) | 344 | (13) | 1905 | (113) |  |
| 10.0 | 1251 | (29) | 336 | (7) | 1603 | (90) |  |
