# Supplementary material for: Production of 13C-labeled docosahexaenoic acid from heterotrophic marine microorganisms Aurantiochytrium mangrovei and Crypthecodinium cohnii enabling fluxomic applications
Source: Front Bioeng Biotechnol. 2025 Nov 19;13:1690863. doi: 10.3389/fbioe.2025.1690863 (PMC12672524; doi:10.3389/fbioe.2025.1690863)
Supplement: Supplementary file 2 [file Table2.docx]

**Table S2** Cellular parameters (size, complexity, lipid content) as measured by flow cytometry on the culture of *C. cohnii* in 40 mL of medium, under control and ^13^C-enriched conditions, values are means ± (SD) (n = 3) (A.U.: arbitrary units, n.d.: not determined)

| **Time (day)** | **Size (A.U.)** | | **Complexity (A.U.)** | | **Lipid Content (A.U.)** | |
| --- | --- | --- | --- | --- | --- | --- |
| **C40-ctrl** | | | | | | |
| 0.0 | 1431 | (0) | 10000 | (0) | n.d. | n.d. |
| 3.8 | 1587 | (22) | 14100 | (346) | 4529 | (710) |
| 5.9 | 1459 | (22) | 13100 | (1418) | 6231 | (866) |
| 7.9 | 1522 | (14) | 10900 | (624) | 6669 | (216) |
| 10.8 | 1623 | (24) | 8730 | (744) | 4336 | (193) |
| 11.7 | 1659 | (33) | 8695 | (389) | 3199 | (289) |
| **C40-13C** | | | | | | |
| 0.0 | 1470 | (0) | 10200 | (0) | n.d. | n.d. |
| 3.8 | 1486 | (10) | 16000 | (265) | 4447 | (21) |
| 5.9 | 1418 | (21) | 13133 | (643) | 8084 | (499) |
| 7.9 | 1515 | (6) | 10767 | (586) | 8215 | (446) |
| 10.8 | 1547 | (18) | 9256 | (294) | 4049 | (526) |
| 11.7 | 1593 | (33) | 9069 | (325) | 3243 | (115) |
